# Clinical Remarks on the Bowel-Affections of Children in the Medical Wards of the Mercy Hospital

**Published:** 1862-09

**Authors:** N. S. Davis

**Affiliations:** Professor of Clinical Medicine, &c.


ARTICLE XXIX.

### CLINICAL REMARKS ON THE BOWEL-AFFECTIONS OF CHILDREN IN THE MEDICAL WARDS OF THE MERCY HOSPITAL.

By N. S. DAVIS, M.D., Professor of Clinical Medicine, &c.

Gentlemen: — In a preceding clinic, I directed your attention to several cases of diarrhœa in children, and described to you the different varieties of bowel-affections met with in general practise; their history and characteristic symptoms; and the causes, both remote or predisposing, and exciting. This morning, I shall make some brief comments on the pathology of these affections, and then give you my views in regard to the principles on which their treatment should be conducted. From the careful review of the causes which are most efficient in producing this class of diseases, and their *modus operandi*, which was made at the preceding clinic, you will readily infer the most important items connected with their pathology. It was then shown that all the causes concerned in the production of the diarrhœa, cholera morbus, and dysentery of young children, co-operated to produce an increased susceptibility or irritability of the mucus membrane of the stomach and bowels, with a diminution of vital affinity, and consequent relaxation of the capillary system of vessels. This morbid excitability, coupled with impaired tonicity of the mucous tissue, constitutes the primary pathological condition in all the first and second groups of cases mentioned in the former lecture. The morbid excitability of the membrane invites a rapid influx of blood into it, while the diminished vital affinity and consequent relaxation of the texture admits of an equally rapid effusion or exudation of the serous part of the blood, and thus furnishes the matter for the copious thin discharges. The rapid loss of the watery element of the blood, carrying with it the salts by this effusion into the stomach and bowels, speedily diminishes all the glandular secretions, such as urine, bile, gastric and salivary juices, etc.; retards organic or molecular changes in all the tissues, thereby diminishing the evolution of caloric; and causes a marked shrinking of the whole body.

The morbid sensibility of the nervous filiments involved in the mucous membrane, acted upon by the effused fluid, calls into action a reflex influence upon the muscular coat, and thereby establishes the frequent efforts to evacuate the stomach and bowels. Such are the pathological conditions which constitute the active stage of these diseases.

They cannot exist long, however, without inducing other pathological changes of no less importance. Thus the continuance of the serous discharges more or less rapidly exhausts the blood of its water and salts, leaving it too viscid to circulate freely through the capillary system of vessels; while the rapidity of effusion through the mucous membrane carries with it more or less of the epithelial cells of that membrane which may be easily detected in the evacuations by the aid of a microscope. If vomiting exists to such an extent as to prevent the retention of drinks long enough to afford any replenishment of the water in the blood, there will be danger of an entire suspension of capillary circulation, and a speedily fatal collapse. Or the increased viscidity of the blood may so modify its relation to the capillaries of the mucous membrane, as to stop the effusion and consequent discharges spontaneously before the state of collapse is reached. If the discharges thus cease before complete collapse ensues, rest and a judicious replenishment of the blood by liquid nourishment, soon restores the patient to health, in the majority of cases; but in some, another pathological state is developed which should never be overlooked. I have said that, during the active stage of diarrhœa or cholera morbus, the rapid efflux of fluid through the mucous membrane carried with it more or less of the epithelial cells of that membrane, and thereby produced more or less impairment of the texture.

Hence it happens that, when the discharges cease, capillary circulation and organic actions are resumed, and the patient is said to have a healthy reaction fairly established, the impairment of texture in some portions of the mucous membrane is such that the capillaries are incapable of resuming their circulatory function. Hence the blood accumulates in them; a low grade of inflammatory action is established in a few hours; and the general reaction passes beyond the healthy standard to that of fever; and, in common professional phraseology, the patient has passed from an attack of serous diarrhœa or cholera morbus to that of secondary enteric or typhoid fever. The asthenic grade of inflammation thus set up, may gradually subside and allow convalescence to be established in from one to three weeks, or it may progress through the successive stages of softening and ulceration, ending in the exhaustion and death of the patient. But the mucous membrane is not the only structure, in which the capillaries may fail to resume their function, when the active discharges cease, and the stage of reaction has come. When the attack has been violent, and the amount of serous discharge so great as to produce a very marked deficiency of water in the blood, the latter may become so altered in its relation to the capillaries of the brain that the circulation becomes too feeble to sustain the function of the cerebral hemispheres. In such cases, though the intestinal discharges may cease, the circulation and warmth be restored to the extremities, and a general appearance of healthy reaction be established, yet the patient passes into a state of more or less complete coma, from which it seldom recovers.

Another, and perhaps more frequent local failure in the resumption of capillary action, is in the kidneys. In the preceding lecture, I told you that one of the early effects of rapid and copious discharges from the mucous surface of the alimentary canal, was a partial or complete suppression of urine. It sometimes happens that when the active intestinal discharges cease, reaction takes place generally, but the secreting structure of the kidneys fails to keep pace with the improvement in other textures. Consequently no urine is secreted, and symptoms of uremic poisoning are speedily developed.

Such are my views of the pathology of the different stages of the first two groups of bowel-affections, described in the preceding clinic. The pathology of the third group of cases, which we described as accompanied by fever from the commencement of the attack, differs in one primary element from the first two. There is the same morbid susceptibility or irritability of the mucous membrane, and consequently the same unnatural influx of blood into it; but the general relaxation of the capillaries, which in the first cases allowed a rapid escape of the fluids constituting serous discharges, does not exist in this group. Consequently, although the efforts at vomiting or purging, or both, may be frequent and severe, yet the amount of fluids actually evacuated is not large, and they are found to consist largely of mucus, thereby indicating the existence of inflammation.

These pathological views afford us clear and definite indications for treatment in each successive stage of these diseases. Thus, in the first stage of those cases characterized by simple serous discharges, either by vomiting or purging, the indications are to allay the morbid irritability of the mucous membrane, and to increase the tone or contractility of the capillaries. To fulfil these, requires the judicious combination of a tonic and anodyne.

Among the best combinations of this kind are the following:

℞.—Aromat. Sulph. Acid, ........................ ʒj.

Sulph. Magnesia, .................................. ʒj.

Tinct. Opii ........................................ ʒj.

Simple Syrup, ..................................... ℥j.

Water, .............................................. ℥j.

Mix, and give to a child one year old, 15 drops every two, three, or four hours, according to the frequency of the discharges.

℞.—Tannate of Quinine, ......................... 4 grs.
Pulv. Opii. ......................................... 1 gr.
White Sugar, .....................................20 grs.

Mix, and divide into eight powders, of which one may be given every three, four, or six hours, to a child of the age just mentioned.

In the early part of mild cases, the use of one or the other of these formula will generally speedily restore the patient to health. But if the attack is more severe, charcterized by, not only frequent serous discharges, but also partial or complete suspension of important secretions, such as urine and bile, we must combine the anodyne with an *astringent* instead of a tonic, and add to both a small dose of some alterative to aid in restoring these important glandular secretions. In such cases, if the vomiting is frequent, and more especially if the matters ejected are sour, I make a solution of bi-carbonate of soda one drachm, and sulphate of morphine one grain in two ounces of water; and of this give from 6 to 15 drops, according to the age of the child, immediately after each act of vomiting. At the same time, give one of the following powders, every three hours, until the discharges cease, viz.:

℞.—Hydrarg. Chlorid. Mite,..................... 3 grs.
Acetas Plumbi, .................................. 3 grs.
Pulv. Opii. ........................................ 1 gr.
Sacchar. Alba, .................................20 grs.

Mix, divide into six powders.

The rule to give whatever medicine is designed to suppress the vomiting, in small doses, *immediately* after each act of vomiting, is one of much practical value.

Vomiting is an act that cannot be perpetuated continuously, but must always occur in paroxysms with an interval of greater or less length between them. Hence, if a dose of medicine is swallowed immediately after a paroxysm of vomiting it will remain in contact with the mucous membrane of the stomach a few minutes, at least, before another effort at vomiting can be performed. During these few minutes, if the medicine is soluble or already in solution, it will gain some effect, both on the nervous filiments and the capillaries of the mucous membrane. And a repetition of the dose immediately after each paroxysm of vomiting, will soon accumulate an effect sufficient to destroy the morbid sensibility, and consequently stop the vomiting. But if we follow the wishes of the patients, and the inclination of almost all nurses, by withholding the medicine after vomiting until the patient has "rested a little," that little period of rest is just sufficient for the muscular coat to regain its contractility, and the mucous coat to pour out a new supply of serous fluid, and consequently the patient is all ready for another paroxysm of vomiting. Now, if the dose of medicine is administered, in nine cases out of ten, it will be rejected almost as quick as swallowed, and the effect is lost.

The same rule is equally important in reference to the use of enemas for aiding in the suppression of diarrhœa or dysentery. They should always be administered as speedily after an evacution as possible, and while the rectum is entirely empty. The longer the enema is delayed after an evacuation, the more mucous or serous fluid has accumulated in the intestine, and the more readily will the introduction of the enema be followed by an immediate expulsion. You thus see, gentlemen, that in the more violent gastric and intestinal affections, success in their treatment depends almost as much on the time and manner of administering medicine as on the kind of medicine chosen.

I have just stated, that in those cases characterized by frequent vomiting of serous fluid and intestinal discharges, thin and destitute of the coloring matter of bile, small and frequent doses of a solution of morphine and soda, with less frequent doses of calomel and acetate of lead, usually constituted the most efficient means for checking the active progress of the disease, and at the same time favoring the restoration of secretion in the more important organs of depuration, as the kidneys and liver. But many cases are met with, in which the discharges, instead of being sour and destitute of the coloring matter of bile, are all bitter and highly colored with the latter fluid, thereby showing a superabundance instead of deficiency of the biliary secretion. In such cases, instead of giving alkalies or alkaline salts and mercurials, all of which tend to increase glandular secretions, I resort directly to the simple combination of anodynes and astringents, as follows:

℞.—Acetas Plumbi, ................................15 grs.
Sulph. Morph. ................................ 1 gr.
Aqua, ........................................ ℥jj.

Mix, and give to a child one year old, 10 drops after every paroxysm of vomiting; or if the vomiting ceases, and the intestinal discharges continue, the same dose may be continued every three or four hours. In using simple anodynes and astringents, it must be remembered that copious intestinal evacuations are almost always accompanied by scantiness of urine, and that the suppression of such evacuations by opium and simple astringents, whether mineral or vegetable, often leaves the kidneys still in a very inactive state. This difficulty can generally be obviated by giving the child a teaspoonful of the following, between each of the doses of opium and acetate of lead:

℞.—Spts. Nit. Dulc.................................. ℥ss.

Acetas Potassa,..................................... ʒjj.

Simple Syrup,..................................... ℥jjss.

Mix. Shake it up when used.

This seldom offends the stomach, and renders efficient aid in restoring a healthy action of the kidneys.

When the acute stage of cholera infantum and serous diarrhœa has passed by, and the disease assumes a chronic form, with rapid emaciation, coolness of the surface and extremities, and the intestinal discharges still thin, with no dysenteric straining, and little or no intermixture of mucus, then I find some one of the following formulæ to afford relief:

℞.—Erigeron Can. herb, ........................... ℥ss.
Tannate of Quinine, ...........................20 grs.
Sulph. Morph...................................... 1 gr.

Mix. Pour on the whole half a pint of boiling water, to make an infusion. When cold, give to a child one year old, a teaspoonful every two, four, or six hours, according to the frequency of the discharges. This combination has the advantage of being moderately diuretic and tonic, while it is efficiently anodyne and astringent, and has been much used by me for several years past.

In mild chronic cases, in which the evacuations show an excess of acid or sourness, the following will often answer the purpose well:

℞.—Fluid Chalk Mixt.................................. ℥jss.
Tinct. Cinnamon, ................................ ℥ss.
Camph. Tinct. Opii................................. ℥j.

Mix, and give from 10 to 30 drops, according to the age of the child, three or four times a day.

In protracted cases, accompanied by an anemic condition of the patient, the liquor ferri nitratis, in suitable doses will be found very valuable.

Five or six drops of this preparation of iron may be given to a child between 12 and 18 months old, at each mealtime, and one of the following powders at bedtime, viz.:

| | |
|---|---|
| ℞.—Tannate of Quinine, | 3 grs. |
| Pulv. Opii. | 1 gr. |
| Hydrarg. cum creta, | 3 grs. |
| Sacchar. Alba, | 20 grs. |

Mix, divide into six powders.

This is substantially the treatment we directed for the several children you examined at the last clinic.

The third group of bowel-affections of children, those which were described as accompanied by fever and mucus in the discharges, involve a true inflammatory condition of mucous membrane, and their treatment involves the same principles as the treatment of enteric fever and dysentery in adults. Hence, it will be more appropriately discussed when we have cases of those diseases before you.

